# The Application of Pediatric Ureteroscope for Seminal Vesiculoscopy

**DOI:** 10.1155/2015/946147

**Published:** 2015-10-20

**Authors:** Shulin Guo, Donghua Xie, Xiangfei He, Chuance Du, Lunfeng Zhu, Xiaolin Deng, Zhongsheng Yang

**Affiliations:** ^1^Department of Urology, The Affiliated Ganzhou City People's Hospital of Nanchang University, Ganzhou, Jiangxi 341000, China; ^2^Linyi City People's Hospital, Linyi, Shandong 276003, China

## Abstract

To describe a novel technique of transurethral seminal vesiculoscopy using a pediatric ureteroscope in the diagnosis and management of persistent hematospermia, a retrospective study was carried out for 20 patients with recurrent hematospermia whom we evaluated and treated using a 6–7.5F (6F front end and 7.5F rear end) pediatric ureteroscope from August 2009 to September 2013. For the 20 patients, the age ranges from 25 to 48 years with a mean age of 36 years. The duration of the hematospermia ranges from 6 to 48 months with a mean duration of 18 months. Transurethral seminal vesiculoscopy was successfully performed in the 20 cases and the mean operative time was 35 min (ranges from 25 to 90 min). Among the 20 patients, 11 patients were found to have seminal vesiculitis, five were with seminal vesicle stone, one was with prostatic utricle stone, one was with prostate cyst, and one was with ejaculatory duct obstruction. The mean follow-up period was 7 months (ranged from 6 to 12 months). Hematospermia in 19 cases disappeared after the surgery and only in one patient the hematospermia recurred 6 months after the surgery. The cure rate was 95%. This study indicated that transurethral seminal vesiculoscopy could be performed easily using a semirigid pediatric ureteroscope with few complications and is an effective therapeutic approach for persistent hematospermia.

## 1. Introduction

Hematospermia is a common disease in andrology. The etiology is quite complex. However, the majority of hematospermia is related to infection in prostate and seminal vesicle. Symptom relief can be achieved for some of the patients after a course of anti-inflammatory therapy. However, symptoms in a small portion of the patients cannot be improved after medication and the hematospermia persists [1, 2]. From August 2009 to September 2013, we evaluated and treated 20 patients with recurrent hematospermia using the pediatric ureteroscopy with satisfactory outcome. We thus reported the results as below.

## 2. Methods

First of all, all the following work was conducted in accordance with the Declaration of Helsinki (1964). The study was conducted with the understanding and the consent of the patients as well as approval from the Ethical Committee of the Affiliated Ganzhou City People's Hospital of Nanchang University, China.

### 2.1. Patients

The study consists of 20 patients with age ranging from 25 to 48 years and a mean age of 36 years. The duration of the hematospermia ranges from 6 to 48 months with a mean duration of 18 months. All these patients presented with intermittent hematospermia. The color in patients with short interval of ejaculation was bright reddish or pinkish. The color in patients with longer interval of ejaculation was dark reddish or coffee colored. Routine and special tests were performed before the surgery. These include CBC, urinalysis, coagulation function index, and prostate fluid routine examination and culture. Per the prostate routine exam, there were 8 cases with elevated WBC count accompanied by decreased lecithin corpuscle. Per the prostate fluid culture, there were 2 cases infected with* E. coli*, 1 case with* Staphylococcus aureus*, and one case with Proteus syndrome. All the patients were found to have normal serum PSA level. Digital rectal exam revealed no abnormality on prostate. Transrectal color Doppler revealed one case with seminal vesicle cyst, 6 cases with inflamed prostate accompanied by calcification, and 5 cases with unilateral or bilateral seminal vesicle stones.

### 2.2. Surgical Procedure

All patients underwent the seminal vesiculoscopy using 6–7.5F pediatric ureteroscope under epidural anesthesia in lithotomy position. Pancystoscopy was performed initially to investigate prostate, bladder, and bilateral ureteral orifices before withdrawing the ureteroscope to the position of verumontanum. Under direct visualization, we initially located the opening of the verumontanum. With the guidance of an epidural catheter and mild flushing with saline, we then entered prostatic utricle for investigation. After careful examination of the prostate utricle, we withdrew the ureteroscope to the opening of the prostate utricle and looked for openings of bilateral ejaculatory ducts outside at 5 and 7 o'clock positions, under continuous saline flushing. Seminal vesicle was then entered under the guidance of the epidural catheter. For patients with unclear ejaculatory duct openings, we used transurethral plasmakinetic resectoscope to resect the verumontanum to expose the openings. Under the direct vision of ureteroscope, we examined carefully if there were congestion or edema on seminal vesicle mucosa, active bleeding or old blood clot, stone, anatomical abnormalities such as cyst, and new growth. We biopsied those lesions suspected to have pathological abnormality. For those small stones, sand-like stones, and old blood clots, we manually removed them using alligator forceps or flushed them out under pressure produced by bolus injection. For those larger stones inside the seminal vesicle, we cleared them by using Holmium laser (power of 2 joules, frequency 15 Hz) to powderize them followed by saline flushing. For seminal vesicle cysts, we also used the Holmium laser to unroof them. In the end, seminal vesicle cavities were irrigated with antibiotic. The same modalities were used for management of other sides of the seminal vesicle. Postoperatively, every patient was placed a Foley catheter and was treated with intravenous antibiotic for 3–5 days.

## 3. Results

**Figure 1:**
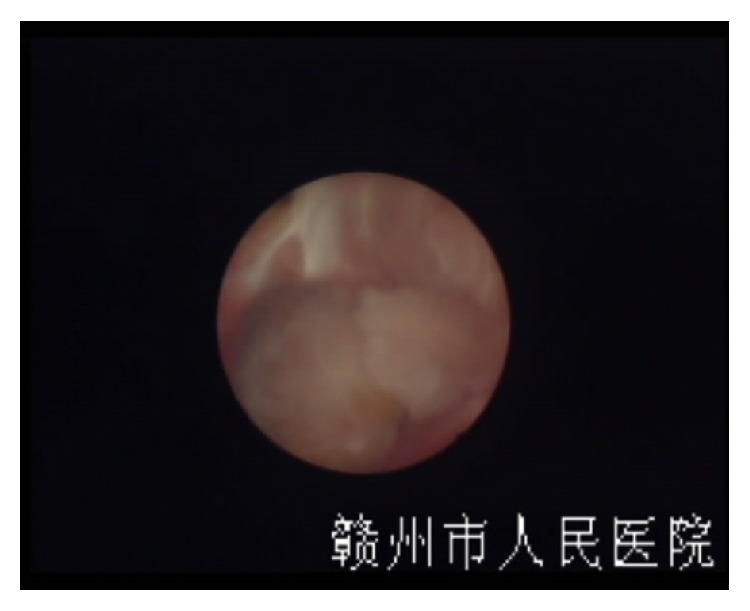
Stone in the cavity of seminal vesicle.

**Figure 2:**
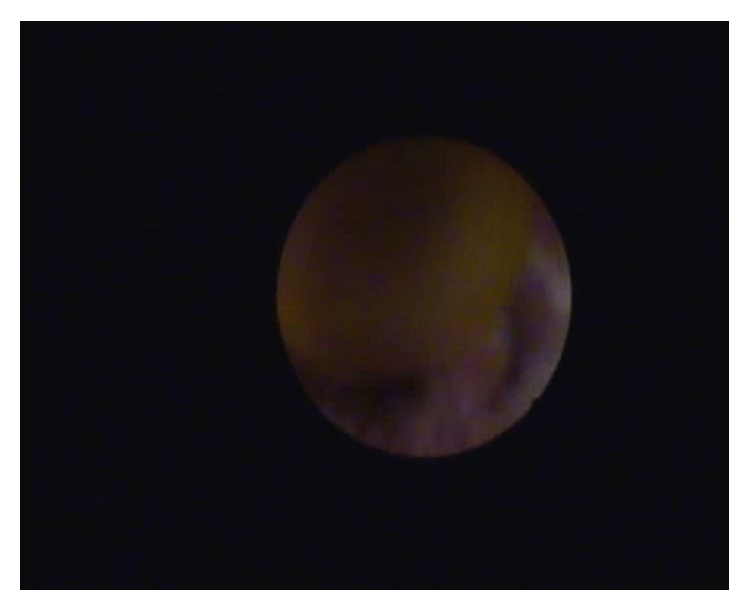
Stone in prostate utricle.

**Figure 3:**
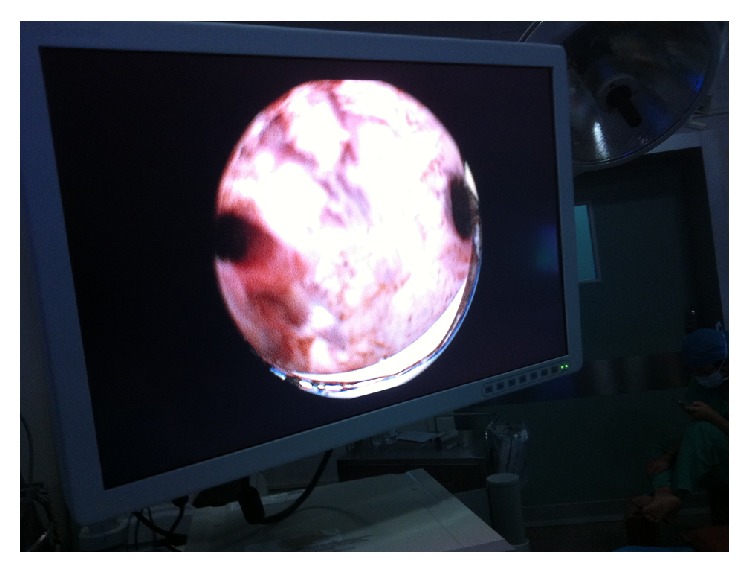
Opened ejaculatory ducts after transurethral plasmakinetic resection.

**Figure 4:**
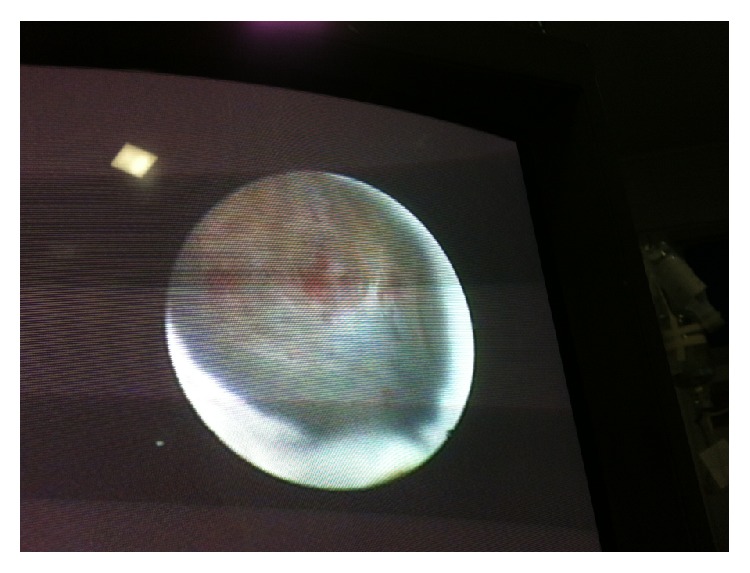
Seminal vesiculitis.

All 20 cases were examined successfully for their seminal vesicles using the pediatric ureteroscopy. The operative time ranged from 25 to 90 minutes with a mean duration of 35 minutes. Eighteen of the 20 cases underwent exam, saline flushing, and antibiotic irrigation bilaterally while for the other 2 cases the exam and treatment were performed unilaterally. In 5 patients there were seminal vesicle stones (left side, 3 cases; right side, 2 cases, Figure 1) which were flushed out after being powderized by holmium laser. In two patients stones were found in prostate utricle (Figure 2), which were extracted manually using alligator forceps. One case of seminal vesicle cyst was unroofed using Holmium laser. One case of ejaculatory duct obstruction underwent transurethral plasmakinetic resection (Figure 3). Eleven cases were found to have edema and congestion in the inner wall of seminal vesicle cavities and a trace of old blood clots, consistent with seminal vesicle wall inflammatory hemorrhage (Figure 4). We followed the cases for 6 to 12 months and found that that there were rare complications. No postoperative epididymitis, retrograde ejaculation, urinary incontinence, and urethral stricture were found in this study. There was one case noticed to have recurrent hematospermia 6 months after surgery with transrectal color Doppler ultrasound revealing a 1.2*∗*0.8 cm left sided para-seminal vesicle cyst, which was improved after a course of anti-inflammatory and physical therapy. The recurrent rate of hematospermia in this series was 5%.

## 4. Discussion

The etiology of hematospermia is quite complex clinically. In general, the etiology can be divided into 3 types including organic, functional, and exigent. The most common circumstance for organic cause is seminal vesicle and prostate inflammation and infection. Other circumstances are ejaculatory duct obstruction, seminal vesicle and prostate stone or cyst, seminal vesicle tuberculosis, and ejaculatory duct injury including iatrogenic factors such as transrectal prostate biopsy [3–9]. In addition, some blood disorders and tumor invasion could also cause hematospermia. The common circumstances for the functional cause are excessive masturbation, excessive sexual intercourse, or too long abstinence time. The exigent cause of the hematospermia could result from minor damage to the ejaculatory duct. Seminal vesicle is the organ to store sperm with abundant vascular layer. When there is inflammatory reaction in the seminal vesicle, the mucosa became congested and edematous and the hematospermia occurs, which often leads to severe stress and fear to the patient [5]. In most patients, the hematospermia may resolve spontaneously in a few weeks or after a course of sensitive antibiotic. However, a small portion of the patients may have recurrent hematospermia resistant to routine therapy, which poses a big challenge for clinical diagnosis and treatment [3, 4].

According to the literature data retrieval, conventional treatment of hematospermia includes systemic administration and local treatment, the latter including prostate massage, hot water bath, physical therapy to improve local blood circulation and promote inflammation absorption and elimination. Due to the special anatomical and physiological characteristics of seminal vesicle and prostate, the treatment efficacy is often poor or not to work for the persistent hematospermia. Therefore, we used the 6–7.5F ureteroscope to investigate the seminal vesicle for those patients with persistent hematospermia, achieving good outcome in both finding out the etiology and delivering therapy.

There are different methods to enter a scope for vesiculoscopy for those patients with unclear ejaculatory duct openings. Some people performed blind insertion through prostate utricle using the scope body forcefully to create a hole, while others used transurethral plasmakinetic resection of verumontanum to expose the ejaculatory duct to enter the scope under direct visualization. We performed one of our 20 cases using the aforementioned blind insertion, which is the case in which recurrent hematospermia was found 6 months after the surgery with a color Doppler ultrasound revealing the left sided para-seminal vesicle cyst. We think this could be caused by seminal vasculitis resulting from blind insertion injury to the ejaculatory duct and incomplete absorption of the intraoperative saline extravasations. But because of the small number of cases and without the blind comparative study, the exact cause of the recurrence needs further investigation.

In our study, we found that the most important causes of hematospermia are inflammation, stenosis, and cyst. Ejaculatory duct stenosis further aggravates the inflammatory reaction. The infection, hemorrhage, and impeded drainage from narrowed ejaculatory duct opening may contribute to the formation of stones, posing a vicious cycle because the stone will further aggravate the stenosis leading to infection and stone formation. For those cases with ejaculatory ducts stenosis with bad exposure of ejaculatory duct opening, we used the transurethral plasmakinetic resection of verumontanum to expose spacious ejaculatory duct openings, not only removed the ejaculatory duct stenosis, but also dredged the ejaculatory duct. The drainage was thus improved, eliminating the root of hematospermia.

Our experiences using the pediatric ureteroscope for seminal vesiculoscopy include the following. (1) The ejaculatory duct openings are most commonly located at 5 and 7 o'clock positions outside the prostate utricle, which allow the 6–7.5F pediatric ureteroscopy to enter under direct visualization in most circumstances. However, it is very important to use epidural catheter as a guide instead of ureteral catheter to avoid edematous effect on the openings and bleeding which will make it difficult to find the openings afterwards. Compared to the ureteral catheter, the epidural catheter is smaller and smoother with better flexibility. Infusing saline through the epidural catheter can facilitate to extend ejaculatory duct openings by maintaining a pressure. (2) It is important to control flushing pressure and speed. We prefer a pressure at 200 mmHg and a speed at 0.2 L/min. (3) We used transurethral plasmakinetic resection of verumontanum instead of traditional vaporization resection for those patients with unclear ejaculatory duct openings, mainly because plasmakinetic resection does not produce coke attachments, reducing the likelihood of postoperative scar stenosis. (4) For patients with intractable hematospermia, we recommend prostate fluid culture and drug susceptibility before the vesiculoscopy and intraoperative irrigation of the seminal vesicle cavities using sensitive antibiotic plus saline.

In summary, we think seminal vesiculoscopy using a pediatric ureteroscope develops a new way to treat refractory hematospermia and plays an important role in promoting the understanding of the cause and treatment of hematospermia. Combining literature reports with our preliminary practical experience, the authors think that the pediatric ureteroscopic examination of seminal vesicle has the advantages of being simple operation, of less trauma, safe, and effective and can be widely used as one of effective methods of diagnosis and treatment of intractable hematospermia. But because of the small number of cases we carried out in the study and the lack of multicenter clinical study of large samples, especially the possible negative thermal effect of holmium laser lithotripsy on mucosa of seminal vesicle by using power of 2 joules and frequency of 15 Hz, the overall and long-term efficacy still needs further observation. Also, we understand that, in case of midline seminal vesicle cyst, we may be able to find the thin lining of mucosa to puncture and introduce the scope to seminal vesicle easily without a need of transurethral resection of verumontanum.
